# Optimized Expression and Purification of Outer Membrane Protein 25 (Omp25) of *Brucella melitensis*

**DOI:** 10.1002/cpz1.70481

**Published:** 2026-10-12

**Authors:** Harshita Mogaveera, Sudipta Patra, Niladri Bhusan Pati, Chiranjay Mukhopadhyay, Manjunath Joshi, Vandana Kalwaje Eshwara

**Affiliations:** ^1^ Department of Microbiology, Kasturba Medical College Manipal Academy of Higher Education Manipal India; ^2^ Department of Microbiology Sapthagiri Institute of Medical Sciences and Research Center Bangalore India; ^3^ Manipal Center for Biotherapeutics Research Manipal Academy of Higher Education Manipal India; ^4^ Department of Ageing Research, Manipal School of Life Sciences Manipal Academy of Higher Education Manipal India

**Keywords:** *B. melitensis*, *E. coli*, expression, Ni‐NTA, recombinant Omp25 protein

## Abstract

Our previous immunoproteomic studies on patients with acute brucellosis identified outer membrane protein 25 (Omp25) of *Brucella melitensis* as one of the immunogenic antigens, indicating its potential to be used as a diagnostic marker and a tool to differentiate acute and chronic human brucellosis. Expression and purification of Omp25 are technically challenging using conventional approaches owing to its membrane association. Existing expression and extraction protocols frequently lead to host cell toxicity, low protein yields, and poor reproducibility. To address these limitations, we developed a modified approach for the expression and purification of recombinant Omp25 protein that is standardized and reproducible and yields protein efficiently. The truncated *omp25* gene is cloned and expressed in *Escherichia coli* BL21 (DE3) cells, and induction is carried out at 28°C for 6 hr. The expressed recombinant Omp25 protein is predominantly found in the insoluble fraction, so it is extracted under denaturing conditions, purified by Ni‐NTA affinity chromatography, refolded by stepwise dialysis, and desalted prior to characterization. In our hands, this set of protocols yielded 47.75 mg purified, high‐quality recombinant Omp25 protein per liter of bacterial culture. SDS‐PAGE analysis demonstrated a single predominant protein band at ∼24.3 kDa with no detectable contaminating protein bands, indicating successful purification. recombinant Omp25–based indirect ELISA confirmed that the refolded protein retained its antigenic properties, exhibiting significantly higher reactivity with sera from culture‐confirmed brucellosis patients than with healthy control sera (p < 0.001). This optimized set of protocols provides a reproducible workflow for the production of highly purified recombinant Omp25 protein and offers quantitative performance metrics that facilitate reproducibility and support this protein's application in immunological and diagnostic studies. © 2026 The Author(s). *Current Protocols* published by Wiley Periodicals LLC.

**Basic Protocol 1**: Expression and extraction of *Brucella melitensis* Omp25 protein from *Escherichia coli* BL21 (DE3) cells

**Basic Protocol 2**: Purification of recombinant Omp25 protein

**Basic Protocol 3**: Refolding and desalting of recombinant Omp25 protein

## INTRODUCTION

Outer‐membrane proteins (OMPs) of Gram‐negative bacteria play an important role in host‐pathogen interaction, immune modulation, and bacterial survival within the host (Koebnik et al., 2000). *Brucella melitensis* is a facultative intracellular pathogen that causes human and animal brucellosis. Previous immunoproteomic studies on patients with acute brucellosis identified outer membrane protein 25 (Omp25) of *B. melitensis* as one of the immunogenic antigens, indicating its potential as a diagnostic marker and a tool to differentiate between acute and chronic human brucellosis (Patra et al., 2025)

Because Omp25 is a membrane‐bound protein, its expression in heterologous systems such as *Escherichia coli* is challenging, often resulting in low solubility, protein aggregation, and inclusion body formation (Sahdev et al., 2008). Existing protein expression and purification protocols have primarily focused on demonstrating successful protein production for immunological and diagnostic applications (Ahmed et al., 2015; Jain et al., 2015; Yang et al., 2020). However, these protocols provide limited quantitative information on protein recovery, purification efficiency, or yield, making direct comparison and protocol standardization difficult. Consequently, there remains a need for a standardized and quantitatively characterized protocol for the efficient expression, purification, and refolding of recombinant Omp25 protein. For the last two decades, various strategies have been developed to overcome the difficulties with the expression and purification of membrane and membrane‐associated proteins. They include removal of signal peptide, optimization of expression temperature and induction conditions, solubilization using chaotropic agents, affinity‐based purification, and controlled refolding approaches (Bornhorst & Falke, 2000; Burgess, 2009; Duong‐Ly & Gabelli, 2014; Middelberg, 2002). Immobilized metal affinity chromatography (IMAC) under denaturing conditions using Ni‐NTA has become a widely used technique for purifying histidine‐tagged proteins that are expressed as inclusion bodies (Bornhorst & Falke, 2000). After purification, refolding is performed by gradual removal of denaturants, and this process has been shown to restore the native conformation and so is suitable for immunodiagnostic applications (Tsumoto et al., 2003). However, differences in buffer systems, denaturant concentration, and refolding strategies often lead to different outcomes across laboratories.

The set of protocols presented here incorporates the existing principles and provides an optimized, stepwise method for the expression of a truncated Omp25 protein (without signal peptide) from *B. melitensis* in *E. coli* BL21 (DE3). This is followed by solubilization and purification of the recombinant protein under denaturing conditions and then refolding of the protein using a suitable buffer. The protocols have been optimized to improve the protein yield, purity, and reproducibility, making this approach suitable for downstream applications. In addition, these protocols can be applied to other difficult‐to‐express bacterial outer membrane or membrane‐associated proteins with similar biochemical properties. The major advantages of this approach are its robustness and adaptability for proteins that are otherwise challenging to purify using conventional methods. For diagnostic applications such as indirect ELISA where antigenicity is the primary requirement, this approach provides a practical and efficient solution.

The entire procedure for recombinant Omp25 protein expression and purification consists of three main parts: (1) expression and extraction of *B. melitensis* Omp25 protein from *E. coli* BL21 (DE3) cells (Basic Protocol 1), (2) purification of the recombinant Omp25 protein (Basic Protocol 2), and (3) refolding and desalting of the recombinant Omp25 protein (Basic Protocol 3).

## Basic Protocol 1 EXPRESSION AND EXTRACTION OF *Brucella melitensis* Omp25 PROTEIN FROM *Escherichia coli* BL21 (DE3) CELLS

Membrane proteins such as Omp25 protein often exhibit poor solubility when they are expressed in heterologous systems. To address this limitation, a truncated form of the *omp25* gene was cloned into the pET28a expression vector using a restriction enzyme‐based cloning strategy. The gene was inserted in such a way that the protein would carry an N‐terminal 6×‐histidine tag, which facilitates downstream purification. This protocol describes an optimized strategy for the expression of truncated Omp25 protein in *E. coli* BL21 (DE3) cells. Following expression, the recombinant Omp25 protein is extracted using a denaturing cell lysis buffer to efficiently recover the expressed protein (Duong‐Ly & Gabelli, 2014; Rosano & Ceccarelli, 2014).

### Materials

*E. coli* BL21 (DE3) cells (Thermo Fisher Scientific, EC0114)
*omp25*‐pET28a plasmid
SOC growth medium (see recipe), 37°C
LB agar with 50 µg/ml kanamycin antibiotic (see recipe)
PCR primers: F: 5′‐TACCGACGCCATCCAGGAAC‐3′, R: 3′‐TAGAACTTGTAGCCGATGCCG‐5′
LB broth with 50 µg/ml kanamycin antibiotic (see recipe)
1 M IPTG stock solution (see recipe)
10 mM sodium phosphate buffer, pH 8.0 (see recipe)
Denaturing cell lysis buffer (see recipe)
Bradford reagent (HiMedia, ML106)
SDS‐PAGE gel (12% resolving gel and 5% stacking gel)
Coomassie Brilliant Blue staining solution (see recipe)
Destaining solution (see recipe)
42°C water bath (BioBee™)
37°C shaker incubator (Rotek)
L‐spreaders, sterile (Tarsons, 920082)
37°C plate incubator (Rotek)
Thermal cycler
Inoculating loops (Tarsons, 920061)
15‐ and 50‐ml conical centrifuge tubes (Tarsons, 546021 and 546041)
Spectrophotometer (BMG Labtech)
100‐ml conical flasks, sterile
Standard tabletop centrifuge (REMI)
Rocking shaker (ROCKYMAX™, Tarsons, 4080)
Probe sonicator (Labman Scientific)
1.5‐ml microcentrifuge tubes (Tarsons, 500010)
4°C microcentrifuge (Eppendorf)

Additional reagents and equipment for *omp25*‐specific PCR, Bradford assay, and SDS‐PAGE (see Current Protocols article: Gallagher, 2012)

*NOTE*: Work should be performed in a class II biosafety cabinet (Esco).

#### Construction of E. coli BL21 (DE3) strain containing omp25‐pET28a vector (Sambrook & Russell, 2001)

1 Thaw competent *E. coli* BL21 (DE3) competent cells and keep them on ice.
2 Add 50 ng (<10 µl) *omp25*‐pET28a plasmid to 200 µl competent *E. coli* BL21 (DE3) cells, gently pipet the suspension to mix, and incubate on ice for 30 min.
  The truncated omp25 gene (without signal peptide) was used for cloning, and the gene was ligated into the pET28a vector using a restriction enzyme–based cloning strategy.
3 Heat‐shock the cell suspension at 42°C for 90 s using a water bath.
4 Immediately place the cell suspension on ice for 1 to 2 min.
5 Add 800 µl SOC growth medium to the cell suspension and incubate at 37°C for 1 hr in a shaker incubator at 200 rpm.
  This step is done for recovery of the transformed cells.
6 Plate 200 µl transformed cell suspension onto LB agar with 50 µg/ml kanamycin antibiotic (final concentration: 50 µg/ml) using sterile L‐spreader.
7 Incubate the plates at 37°C for 16 to 24 hr.
8 Post‐incubation, note down the number of transformants and screen the transformants for the presence the *omp25* gene using *omp25*‐specific PCR primers and a thermal cycler under the following conditions:

**Table:** 

Initial step:	5 min	95°C	(denaturation)
30 cycles:	30 s	94°C	(denaturation)
	30 s	62.4°C	(annealing)
	45 s	72°C	(extension)
Final step:	5 min	72°C	(extension)

#### Expression of recombinant Omp25 protein (Wen‐xing et al., 2011)

9 Pick the positive transformant colony from the agar plate using an inoculating loop and inoculate into 10 ml LB broth with 50 µg/ml kanamycin antibiotic in a 50‐ml conical centrifuge tube.
10 Incubate the suspension overnight at 37°C in a shaker incubator at 150 rpm.
  This step entails preparation of the starter culture.
11 Prepare fresh cultures by inoculating 1% (150 µl) of the overnight culture into 15 ml fresh LB broth with 50 µg/ml kanamycin in a sterile 100‐ml conical flask and incubate at 37°C in a shaker incubator at 150 rpm for 2 hr.
12 Monitor the cell growth with a spectrophotometer until it reaches OD_600_ of 0.6 and then reduce the incubator temperature to 28°C.
13 Once the temperature has reached 28°C, induce protein expression by adding 1.5 µl of 1 M IPTG stock solution (final concentration: 0.1 mM) to culture and incubate for 4 hr in the shaker incubator at 28°C and 150 rpm.
  This set is known as induced culture.
14 Maintain another set of cultures for the same duration but without the addition of IPTG. In addition, collect an uninduced culture immediately in a 15‐ml conical centrifuge tube and centrifuge 10 min at 3500 rpm. Collect the resulting cell pellet and store at –20°C until further processing.
  This set is known as uninduced culture.
15 After 4 hr of induction, collect 15 ml of each induced and uninduced culture in a 15‐ml conical centrifuge tube and centrifuge 10 min at 1600 × *g*. Collect the resulting cell pellet and store at –20°C until further processing.

#### Extraction of recombinant Omp25 protein (Koyuncu et al., 2018; Wen‐xing et al., 2011)

16 Wash the pellet with 10 mM sodium phosphate buffer (pH 8.0) and centrifuge the suspension for 20 min at 1600 × *g*.
17 Remove the supernatant and resuspend the washed pellet in 10 ml denaturing cell lysis buffer.
  A total of 10 ml buffer is used for 100 mg cell pellet.
18 Incubate the cell lysate at room temperature for 30 min on a rocking shaker.
19 Using a probe sonicator, sonicate the cell lysate on ice at 38% range with cycles of 10‐s impulse and 10‐s pause for a total duration of 4 min. Once sonicated, aliquot the cell lysate into 1.5‐ml microcentrifuge tubes.
20 Centrifuge the cell lysate for 30 min at 13,000 rpm, 4°C.
21 Collect the supernatant, containing soluble recombinant Omp25 protein.
22 Estimate the protein concentration using Bradford assay and analyze the protein expression by SDS‐PAGE using an SDS‐PAGE gel (12% resolving gel and 5% stacking gel), Coomassie Brilliant Blue staining solution, and destaining solution.
  A representative SDS‐PAGE gel image of successful protein extraction under the optimized induction conditions mentioned above is shown in Figure 1.

**Figure 1:**
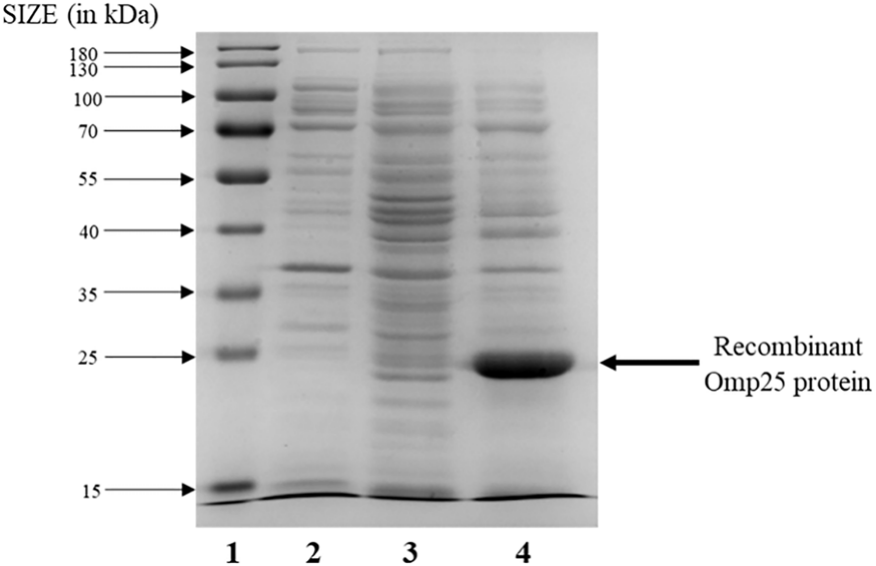
SDS‐PAGE gel image of expression carried out at 28°C for 4 hr, with induction with 0.1 mM IPTG. Cell lysis was performed using denaturing cell lysis buffer. 1: Protein ladder (Thermo Fisher Scientific, 26616), 2: Uninduced (0 hr), 3: Uninduced (4 hr), 4: Induced with 0.1 mM IPTG (4 hr).

## Basic Protocol 2 PURIFICATION OF RECOMBINANT Omp25 PROTEIN

The extracted recombinant His_6_‐Omp25 protein (Basic Protocol 1) is purified using Ni‐NTA affinity chromatography. This approach uses the interaction between the histidine residues in the protein and the nickel ions on the column. This interaction enables purification of the target protein (His_6_‐Omp25 protein). The protocol describes purification of the His_6_‐Omp25 protein under denaturing conditions (Koyuncu et al., 2018; Wen‐xing et al., 2011).

### Materials

Protein extract obtained after cell lysis (see Basic Protocol 1)
Equilibration buffer (see recipe)
Wash buffer (see recipe)
Elution buffer (see recipe)

HisPur™ Ni‐NTA spin columns (Thermo Fisher Scientific, 88224)
15‐ml conical centrifuge tubes (Tarsons, 546021)
Standard tabletop centrifuge (REMI)
Rocking shaker (ROCKYMAX™, Tarsons, 4080)

Additional reagents and equipment for SDS‐PAGE (see Current Protocols article: Gallagher, 2012) and Bradford assay (see also Basic Protocol 1)

1 Remove the HisPur™ Ni‐NTA spin columns from 2° to 8°C storage and place at room temperature.
2 Prepare the protein extract obtained after cell lysis by mixing 1 ml protein extract with 1 ml equilibration buffer.
3 Remove the bottom closure of the Ni‐NTA spin column and place the column into 15‐ml conical centrifuge tube.
4 Centrifuge the column for 2 min at 700 *g* and discard the storage buffer.
5 Equilibrate the column by adding 2 ml equilibration buffer and allow the buffer to enter the resin bed, followed by centrifugation for 2 min at 700 × *g*. Discard the buffer.
6 Load the prepared protein sample (see step 2) and allow it to enter the resin bed.
7 Incubate the column for 30 min at room temperature on rocking shaker.
  This incubation step will help in the optimal binding of the His_6_‐Omp25 to the resin.
8 Centrifuge the column for 2 min at 700 × *g* and collect the flow‐through fraction (FT1) in a 15‐ml conical centrifuge tube.
9 Wash the column with 2 ml wash buffer, followed by centrifugation for 2 min at 700 × *g*, and collect the wash fraction (W1) in a 15‐ml conical centrifuge tube.
10 Repeat the washing step three additional times and collect the wash fractions as W2, W3, and W4 in 15‐ml conical centrifuge tubes.
11 Elute the bound protein by adding 1 ml elution buffer, followed by centrifugation for 2 min at 700 × *g*, and collect the elution fraction (E1) in a 15‐ml conical centrifuge tube.
12 Repeat the elution step two additional times and collect the elution fractions as E2 and E3 in 15‐ml conical centrifuge tubes.
13 Analyze all collected fractions by SDS‐PAGE and Bradford assay.
  A representative SDS‐PAGE gel image of successful Ni‐NTA purification under denaturing conditions is shown in Figure 2, where the eluted fraction has a band at ∼25 kDa.

**Figure 2:**
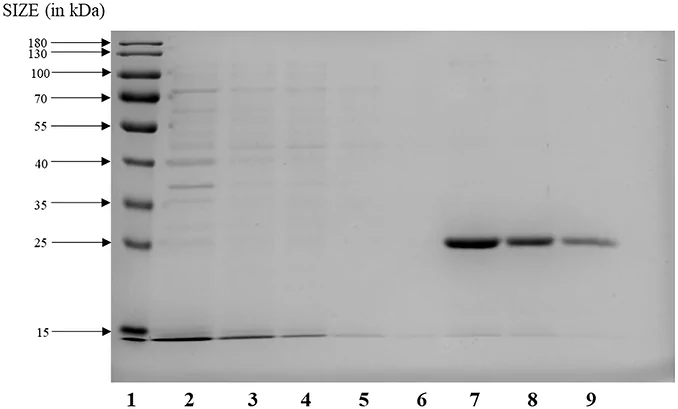
SDS‐PAGE gel image of extracts after Ni‐NTA purification. 1: Protein ladder (Thermo Fisher Scientific, 26616), 2: FT1, 3: W1, 4: W2, 5: W3, 6: W4, 7: E1, 8: E2, 9: E3.

## Basic Protocol 3 REFOLDING AND DESALTING OF RECOMBINANT Omp25 PROTEIN

The denatured His_6_‐Omp25 protein obtained from Ni‐NTA purification (Basic Protocol 2) is refolded to its near‐to‐native conformation using dialysis. The purpose of refolding the His_6_‐Omp25 protein is to incorporate it as an antigen in indirect ELISA, in which it is ideal to use the protein in its native conformation. Refolding is carried out under controlled buffer conditions to allow the gradual removal of urea from the protein extract. This removal will facilitate folding of the His_6_‐Omp25 protein (Singh & Panda, 2005; Sørensen & Mortensen, 2005). After refolding, the protein extract is subjected to desalting to remove the residual small salt molecules (<14 kDa), which yields protein extract suitable for downstream applications. Following purification and refolding, the antigenicity of the recombinant Omp25 protein was evaluated by indirect ELISA using sera from culture‐confirmed brucellosis patients (n = 10) and healthy individuals (n = 10).

### Materials

Eluted His_6_‐Omp25 protein extracts (see Basic Protocol 2)
20 mM sodium phosphate buffer, pH 7.4 (see recipe), room temperature and 4°C
2 M, 4 M, and 6 M urea dialysis buffers (see recipes), 4°C
Urea‐free dialysis buffer (see recipe)

Dialysis tubing cellulose membrane (Sigma‐Aldrich, D9777)
250‐ml borosilicate glass beaker
Dialysis tubing closures (Sigma‐Aldrich, Z370991)
Magnetic stirrer (Tarsons, 4030)
Magnetic stirrer bar (Tarsons, 4141)
15‐ml conical centrifuge tubes (Tarsons, 546021)
Zeba™ Spin Desalting Columns (Thermo Fisher Scientific, 89889)
Standard tabletop centrifuge (REMI)

#### Refolding of recombinant Omp25 protein using dialysis (Koyuncu et al., 2018)

1 Pool the eluted His_6_‐Omp25 protein extracts.
  The pooled extracts will be subjected to dialysis to gradually remove the urea.
2 Cut 10 cm of dialysis tubing cellulose membrane.
3 Keep the membrane in 200 ml of cold 20 mM sodium phosphate buffer (pH 7.4) in a 250‐ml borosilicate glass beaker for 3 to 4 hr at 4°C.
  This will help in activation/equilibration of the dialysis membrane.
4 Seal one side of the dialysis membrane using dialysis tubing closures.
5 Transfer the pooled eluted protein extracts (see step 1) into activated dialysis tubing and securely seal the open end.
6 Place the dialysis bag in cold 6 M urea dialysis buffer and incubate for 2 to 3 hr at 4°C with gentle stirring on magnetic stirrer with a magnetic stirrer bar.
7 Replace the buffer with cold 4 M urea dialysis buffer followed by cold 2 M urea dialysis buffer, incubating for 2 to 3 hr each at 4°C with gentle stirring.
8 Perform final dialysis overnight against urea‐free dialysis buffer to allow complete refolding of the recombinant Omp25 protein.
9 Remove the seal and collect the refolded protein sample into a fresh 15‐ml conical centrifuge tube.

#### Desalting of recombinant Omp25 protein extract using size‐exclusion chromatography

10 Twist off the Zeba™ Spin Desalting Column's bottom closure and loosen the cap. Place the column in 15‐ml conical centrifuge tube.
  The residual imidazole and other salts from the protein extracts will be removed using the Zeba™ Spin Desalting columns.
11 Centrifuge the column for 2 min at 1000 × *g* and remove the storage solution.
12 Place a mark on the side of the column where the compacted resin is slanted upward.
  Place the column in centrifuge with the mark facing outward in all subsequent centrifugation steps.
13 Add 1 ml of 20 mM sodium phosphate buffer (pH 7.4) to the column.
14 Centrifuge 2 min at 1000 × *g* to remove the buffer.
15 Repeat the steps 15 and 16 two or three additional times and discard the buffer from the collection tube.
16 Place the equilibrated column in a new collection tube, remove the cap, and slowly load 700 µl protein sample from step 9 onto the center of the compact resin bed.
17 Add 40 µl of 20 mM sodium phosphate buffer (pH 7.4) to the column.
  This is done to ensure maximal protein recovery.
18 Centrifuge 2 min at 1000 × *g* to collect the protein extract.

## REAGENTS AND SOLUTIONS

### Base buffer for Ni‐NTA purification

24.02 g urea (HiMedia, TC180)
500 µl Triton‐X‐100 (Sigma‐Aldrich, T8787)
50 ml 1× phosphate‐buffered saline (PBS), pH 7.4 (see recipe)
Store ≤1 month at 2° to 8°C

### Coomassie Brilliant Blue staining solution

0.25 g Coomassie Brilliant Blue R‐250 (HiMedia, MB153)
Dissolve in 100 ml destaining solution (see recipe)
Filter using Whatman No. 1 filter paper
Store ≤1 year at room temperature

### Destaining solution

250 ml methanol (Sigma‐Aldrich, 179337‐500ML)
50 ml glacial acetic acid (Sigma‐Aldrich, A6283)
200 ml distilled H_2_O
Store ≤1 year at room temperature

### Denaturing cell lysis buffer

100 ml 20 mM sodium phosphate buffer, pH 8.0 (see recipe)
Add 48.05 g urea (HiMedia, TC180) (final concentration: 8 M)
Mix at 35°C for 15 to 20 min until urea dissolves
Add 2.92 g NaCl (final concentration: 0.5 M)
Add 200 µl 10 mg/ml lysozyme stock solution (see recipe)
Add 200 µl protease‐inhibitor cocktail (Sigma‐Aldrich, P8849)
2 µl nucleases (Thermo Fisher Scientific, 88700)
Mix well
Store ≤1 month at 2° to 8°C

### Elution buffer

1.5 ml 2 M imidazole solution (provided with HisPur™ Ni‐NTA spin columns, Thermo Fisher Scientific, 88224) (final concentration: 250 mM)
8.5 ml base buffer for Ni‐NTA purification (see recipe)
Store ≤1 month at 2° to 8°C

### Equilibration buffer

50 µl 2 M imidazole solution (provided with HisPur™ Ni‐NTA spin columns, Thermo Fisher Scientific, 88224) (final concentration: 10 mM)
9.950 ml base buffer for Ni‐NTA purification (see recipe)
Store ≤1 month at 2° to 8°C

### IPTG stock solution, 1 M

2.383 g isopropyl β‐D‐thiogalactopyranoside (IPTG; HiMedia, MB072‐5G)
10 ml autoclaved distilled H_2_O
Mix well
Filter using 0.22‐µm syringe filter
Mix well
Prepare 100‐µl aliquots in microcentrifuge tubes
Store ≤1 year at –20°C

### Kanamycin stock solution, 50 mg/ml

500 mg kanamycin powder (Sigma‐Aldrich, 60615)
10 ml autoclaved distilled H_2_O
Mix well
Prepare 100‐µl aliquots in microcentrifuge tubes
Store ≤1 year at –80°C
  Do not freeze‐thaw the antibiotic; use one antibiotic vial once.

### LB agar with 50 µg/ml kanamycin antibiotic

2.5 g Luria‐Bertani (LB) broth powder (HiMedia, M1245)
100 ml distilled H_2_O
2.5 g agar (HiMedia, GRM026)
Autoclave
Cool to 45° to 50°C
Add 100 µl 50 mg/ml kanamycin stock solution (recipe)
Pour 20 ml medium into each Petri plate (gamma‐irradiated Petri plates, Abdos, P10909)
Let solidify
Store ≤15 days at 2° to 8°C

### LB broth

2.5 g LB broth powder (HiMedia, M1245)
100 ml distilled H_2_O
Autoclave
Cool to 45° to 50°C
Store ≤1 month at 2° to 8°C

### LB broth with 50 µg/ml kanamycin antibiotic

100 ml sterile LB broth (see recipe)
Add 10 µl 50 mg/ml kanamycin stock solution (see recipe)
Mix well
Store ≤1 week at 2° to 8°C

### Lysozyme stock solution, 10 mg/ml

100 mg lysozyme powder (Sigma‐Aldrich, 62971‐10G‐F)
10 ml 10 mM Tris·Cl, pH 8.0 (see recipe)
Mix well
Filter using 0.22‐µm syringe filter
Store ≤1 week at 2° to 8°C

### Phosphate‐buffered saline (pH 7.40, 1×

Dissolve 1 tablet PBS (Sigma‐Aldrich, P4417) in 200 ml autoclaved distilled H_2_O
Store ≤1 month at 2° to 8°C

### SOC growth medium

2 g SOC medium (HiMedia, G015‐500g)
100 ml distilled H_2_O
Autoclave
Cool to 45° to 50°C
Store ≤1 month at 2° to 8°C
  SOC medium is also known as Super Optimal broth with Catabolite repression medium.

### Sodium phosphate buffer (pH 7.4), 20 mM

0.87 g Na_2_HPO_4_ anhydrous (HiMedia, GRM1416)
0.54 g NaH_2_PO_4_.H_2_O (Merck, 1.06346)
500 ml autoclaved distilled H_2_O
Mix well
Store ≤1 month at 2° to 8°C

### Sodium phosphate buffer (pH 8.0), 10 mM

19 mg NaH_2_PO_4_.H_2_O (Merck, 1.06346)
0.122 g Na_2_HPO_4_ anhydrous (HiMedia, GRM1416)
100 ml autoclaved distilled H_2_O
Mix well
Check that pH is 8.0
Store ≤1 month at 2° to 8°C

### Sodium phosphate buffer (pH 8.0), 20 mM

38 mg NaH_2_PO_4_.H_2_O (Merck, 1.06346)
0.245 g Na_2_HPO_4_ anhydrous (HiMedia, GRM1416)
100 ml autoclaved distilled H_2_O
Mix well
Check that pH is 8.0
Store ≤1 month at 2° to 8°C

### Tris·Cl (pH 8.0), 10 mM

0.121 g Tris buffer (SRL Chemicals, 37969)
Dissolve in 80 ml autoclaved distilled H_2_O
Adjust pH to 8.0 using 4 N HCl
H_2_O to 100 ml
Store ≤1 month at 2° to 8°C

### Urea dialysis buffer, 2 M

12.01 g urea (HiMedia, TC180)
100 ml dialysis buffer (see recipe)
Mix at 35°C for 15 to 20 min until urea dissolves
Store ≤1 month at 2° to 8°C

### Urea dialysis buffer, 4 M

24.02 g urea (HiMedia, TC180)
100 ml dialysis buffer (see recipe)
Mix at 35°C for 15 to 20 min until urea dissolves
Store ≤1 month at 2° to 8°C

### Urea dialysis buffer, 6 M

36.36 g urea (HiMedia, TC180)
100 ml dialysis buffer (see recipe)
Mix at 35°C for 15 to 20 min until urea dissolves
Store ≤1 month at 2° to 8°C

### Urea‐free dialysis buffer

500 µl Triton‐X‐100 (Sigma‐Aldrich, T8787)
43.6 g L‐arginine (HiMedia, TC052)
0.307 g reduced glutathione (Sigma‐Aldrich. G4251)
0.0305 g oxidized glutathione (Sigma‐Aldrich, G4501)
500 ml 20 mM sodium phosphate buffer, pH 7.4 (see recipe)
Mix well
Store ≤1 month at 2° to 8°C

### Wash buffer

100 µl 2 M imidazole solution (provided with HisPur™ Ni‐NTA spin columns, Thermo Fisher Scientific, 88224) (final concentration: 20 mM)
9.9 ml base buffer for Ni‐NTA purification (see recipe)
Store ≤1 month at 2° to 8°C

## COMMENTARY

### Critical Parameters

#### Induction conditions (Basic Protocol 1)

• For induction, use *E. coli* BL21 (DE3) cells at mid‐log phase, i.e., OD_600_ of 0.5 to 0.6. Adding IPTG outside this range can reduce expression efficiency or increase cellular stress, especially affecting membrane‐associated proteins such as Omp25.

• Add IPTG after lowering the temperature to 28°C to minimize protein aggregation and host cell toxicity.

#### Cell lysis parameters (Basic Protocol 1)

• Dissolve the urea at 35°C; do not exceed this temperature, as it may lead to degradation.

• Adding protease inhibitor cocktail during lysis is essential to prevent degradation of the recombinant Omp25 protein.

• Perform sonication on ice, as during the process, the temperature of the solution will rise, which could lead to protein denaturation.

#### Purification parameters (Basic Protocols 2 and 3)

• Ni‐NTA purification should be performed using urea‐containing buffer, as its absence may lead to precipitation of the proteins.

• Additionally, dialysis should be done very slowly as rapid removal of urea may lead to irreversible aggregation of the Omp25 protein.

• Use chilled buffers while performing dialysis.

#### Other parameters

• Store the protein samples at –20°C and avoid freeze‐thawing, which may lead to denaturation of the proteins and aggregation.

### Troubleshooting

Please see Table 1 for a troubleshooting guide.

**Table 1:** Troubleshooting Guide for Expression, Extraction, and Purification of Recombinant Omp25 Protein

Problem	Possible cause(s)	Solution
Low or no expression of recombinant Omp25	Host cell toxicity and death due to membrane protein expression	Remove signal peptide from the protein and express it in *E. coli* BL21 (DE3) cells
	Induction was not done at mid‐log phase	Induce culture at OD_600_ of 0.5‐0.6 for optimal expression
	Host cell toxicity due to membrane protein expression	Reduce induction temperature or time
Poor solubilization of cell pellet	Insufficient denaturant concentration	Ensure lysis buffer contains 8 M urea throughout extraction
	Incomplete cell lysis	Optimize sonication parameters and ensure adequate lysozyme treatment
Highly viscous lysate	Incomplete nucleic acid degradation	Increase nuclease concentration or incubation time during lysis
Protein degradation	Freeze‐thaw cycles	Prepare protein in aliquots and use each aliquot only once
	Lack of protease inhibitors	Add fresh protease inhibitors
Poor binding of protein to Ni‐NTA column	Incorrect buffer composition	Maintain denaturing conditions throughout the purification
	Incorrect pH	Maintain pH around 7.5‐8.0
Low protein recovery after elution	Insufficient imidazole concentration in elution buffer	Optimize imidazole concentration in elution buffer
Protein precipitation during dialysis	Rapid removal of urea	Gradually remove urea
Low solubility of refolded protein	Suboptimal refolding conditions	Adjust pH or ionic strength of the dialysis buffer or include stabilizing agents

### Understanding Results

The key optimizations in the presented approach include induction conditions, inclusion‐body solubilization using 8 M urea in lysis buffer, Ni‐NTA purification under denaturing conditions, and stepwise refolding using L‐arginine and a glutathione redox system. These improvements enhance protein recovery and reproducibility while preserving antigenic reactivity, addressing several limitations of previously published methods for recombinant Omp25 protein production (Table 2).

**Table 2:** Comparison of the Present Recombinant Omp25 Expression and Purification Approach with Previously Published Methods

Study	Host strain	Expression conditions	Purification	Quantitative yield reported	Protein characterization
Jain et al., 2015	*E. coli* BL21	0.6 mM IPTG induction	Ni‐NTA	Not reported	Western blotting
Ahmed et al., 2015	*E. coli* BL21 (DE3)	Auto‐induction	Ni‐NTA	Not reported	Western blotting
Yang et al., 2020	*E. coli* BL21 (DE3)	1 mM IPTG induction	Ni‐NTA	Not reported	SDS‐PAGE
Present study	*E. coli* BL21 (DE3)	Optimized IPTG concentration, induction temperature, and duration	Denaturing Ni‐NTA + dialysis/desalting	24.4% post‐dialysis recovery	Western blotting and indirect ELISA

Unlike above‐mentioned studies, which primarily focused on SDS‐PAGE for the characterization of recombinant Omp25 protein, the presented approach can additionally provide quantitative data on soluble protein, affinity purification recovery, post‐dialysis recovery, and protein yield per liter of bacterial culture, thereby enabling a more comprehensive evaluation of the purification process.

In our hands, from 15 ml induced *E. coli* culture, ∼135 mg wet cell pellet was obtained. Cell lysis yielded 11.88 mg total soluble protein (1188 µg/ml), of which 5.94 mg (5 ml) was subjected to Ni‐NTA affinity purification using five 1‐ml columns. The pooled first elution (5 ml) contained 2.93 mg purified protein (586 µg/ml), corresponding to an apparent recovery of 49.3% relative to the total protein loaded onto the Ni‐NTA columns. Following dialysis and desalting, the purified protein concentration was 143.25 µg/ml in a final volume of 5 ml, yielding 716.25 µg purified protein. This corresponded to a 24.4% recovery following dialysis/desalting and an overall recovery of 12.1% relative to the total protein loaded onto the Ni‐NTA columns, with a final yield of ∼47.75 mg purified protein per liter of bacterial culture.

Furthermore, SDS‐PAGE analysis of the refolded protein showed a single predominant band of ∼24.3 kDa, with no detectable contaminating protein bands, indicating successful purification of the recombinant Omp25 protein (Fig. 3). Recombinant Omp25‐based indirect ELISA showed significantly higher reactivity with sera from culture‐confirmed brucellosis patients (n = 10) than sera from healthy controls (p < 0.001), indicating that the refolding process successfully preserved the antigenically relevant epitopes of recombinant Omp25 protein (Fig. 4).

**Figure 3:**
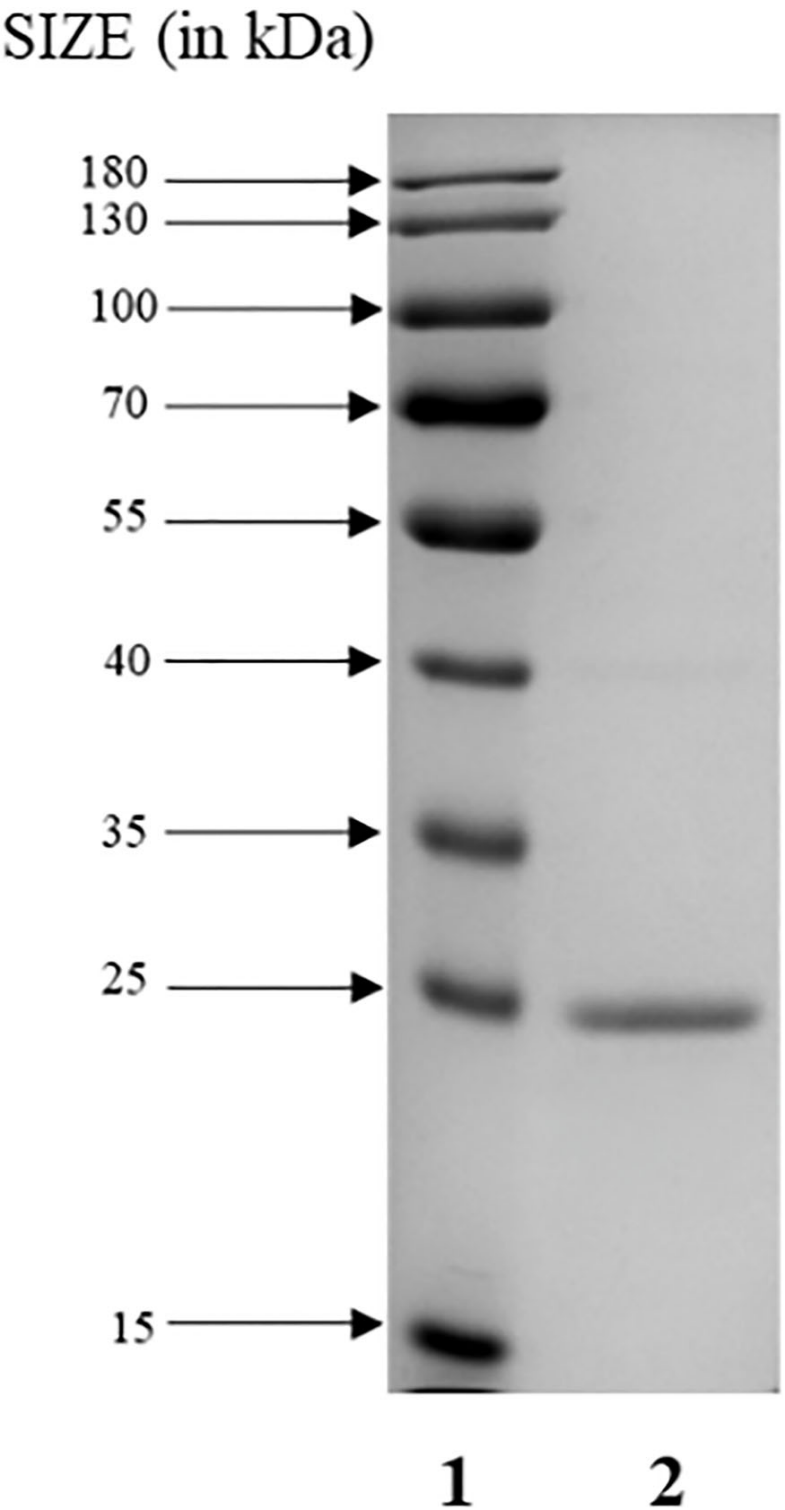
SDS‐PAGE gel image of refolded and desalted recombinant Omp25 protein. 1: Protein ladder (Thermo Fisher Scientific, 26616), 2: Refolded and desalted recombinant Omp25 protein.

**Figure 4:**
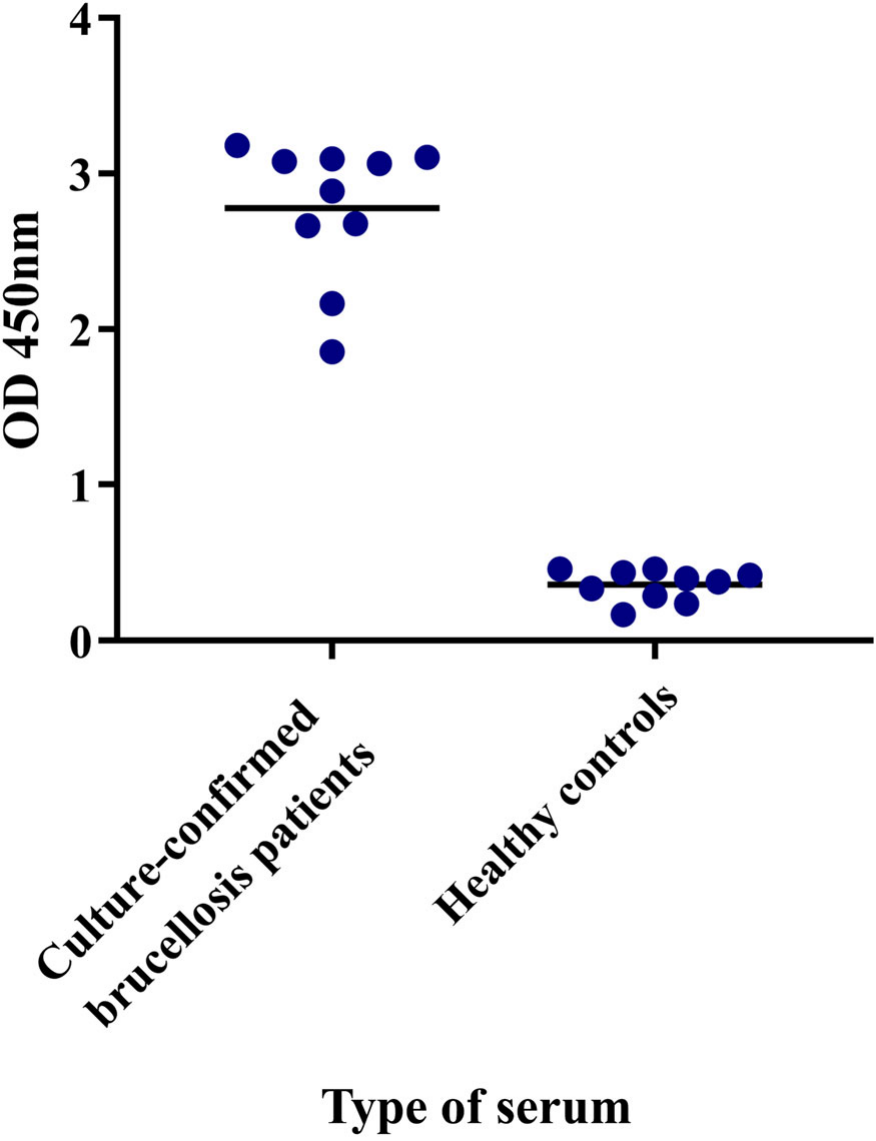
Indirect ELISA reactivity of recombinant Omp25 protein with sera from culture‐confirmed brucellosis patients (n = 10) and healthy controls (n = 10).

Overall, the optimized workflow provides a reproducible method for producing highly purified recombinant Omp25 protein with quantitative recovery metrics and preserved immunoreactivity, making it suitable for downstream diagnostic applications.

### Time Considerations

Following are the time considerations for each protocol.

#### Basic Protocol 1

Media preparation, autoclaving, and pouring will take 2 to 3 hr.

Setting up the transformation reaction will take 2 hr plus overnight incubation of the plates.

Screening for transformants and sub‐culturing the transformants will take 1 to 2 days.

For expression, the starter culture needs overnight incubation, followed by induction and harvesting cells, which will take 6 to 8 hr.

Buffer preparation and cell lysis will take ∼2 days.

#### Basic Protocol 2

Purification using Ni‐NTA columns will take 3 to 4 hr if the buffers are already prepared.

#### Basic Protocol 3

Refolding and desalting will take 2 to 3 days.

Analysis of the expression of recombinant protein using SDS‐PAGE will take 3 days (including buffer preparation).

### Author Contributions

**Harshita Mogaveera**: Conceptualization; data curation; formal analysis; investigation; methodology; software; validation; visualization; writing—original draft. **Sudipta Patra**: Conceptualization; data curation; funding acquisition; methodology. **Niladri Bhusan Pati**: Writing—original draft; writing—review and editing. **Chiranjay Mukhopadhyay**: Conceptualization; investigation; methodology; validation; writing—review and editing. **Manjunath Joshi**: Investigation; supervision; validation; writing—review and editing. **Vandana Kalwaje Eshwara**: Conceptualization; data curation; formal analysis; funding acquisition; investigation; methodology; project administration; supervision; writing—original draft; writing—review and editing.

### Conflict of Interest

No conflicts of interest are declared.

## Data Availability

The data that support the protocols are available from the corresponding author upon reasonable request.
